# 216. Effectiveness of JYNNEOS Vaccination in Preventing Lesion Dissemination Among Individuals with Confirmed Mpox Infection

**DOI:** 10.1093/ofid/ofae631.074

**Published:** 2025-01-29

**Authors:** Lauren Granskog, Kayla Saadeh, Robert Snyder, Eric C Tang, Timothy Lo, Joshua Quint, Tarek Salih, Kathleen Jacobson, Joseph Lewnard, Kieran Lorenz, Marisa Ramos

**Affiliations:** University of California Berkeley, Berkeley, CA; California Department of Public Health, Richmond, California; California Department of Public Health, Richmond, California; Sexually Transmitted Diseases Control Branch, Richmond, California; California Department of Public Health, Richmond, California; California Department of Public Health, Richmond, California; California Department of Public Health, Richmond, California; CDPH, Richmond, California; University of California Berkeley, Berkeley, CA; California Department of Public Health, Richmond, California; California Department of Public Health, Richmond, California

## Abstract

**Background:**

Observational studies during the global mpox clade IIb outbreak associated with male-to-male sexual contact have demonstrated the JYNNEOS vaccine prevents diagnosed mpox disease. However, effects of vaccination on mpox clinical presentations remain uncertain.

Data Flow Chart

**Figure:**
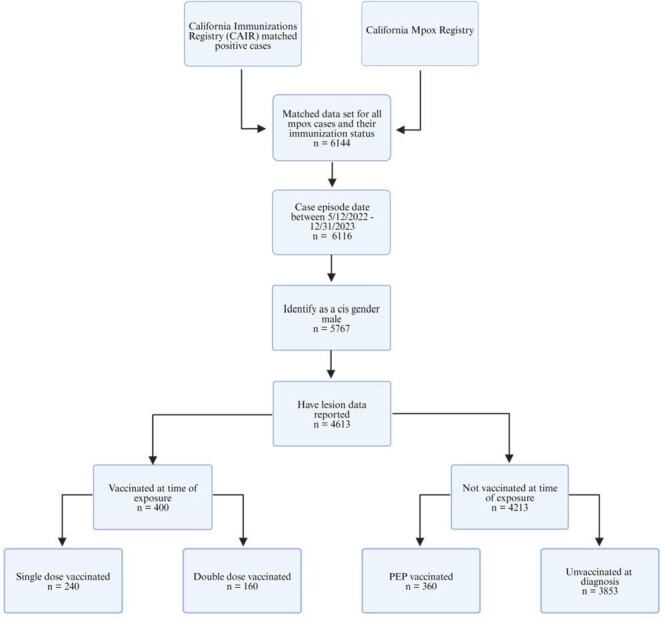

Flow chart characterizing the mpox cases in California’s eligibility for inclusion in the present analysis in relation to their genders, clinical presentations, and vaccination statuses.

**Methods:**

Male cases with confirmed mpox infection reported to the California Department of Public Health between May 12, 2022 and December 31, 2023 were interviewed about anatomical lesion sites. We ascertained cases’ vaccination statuses via linkage to the California Immunization Registry. We estimated JYNNEOS vaccine effectiveness (VE) against progression to disseminated disease via the adjusted odds ratio of vaccination among cases with lesions across multiple anatomical regions versus cases with lesions contained to a single anatomical region.

Body Regions

**Figure:**
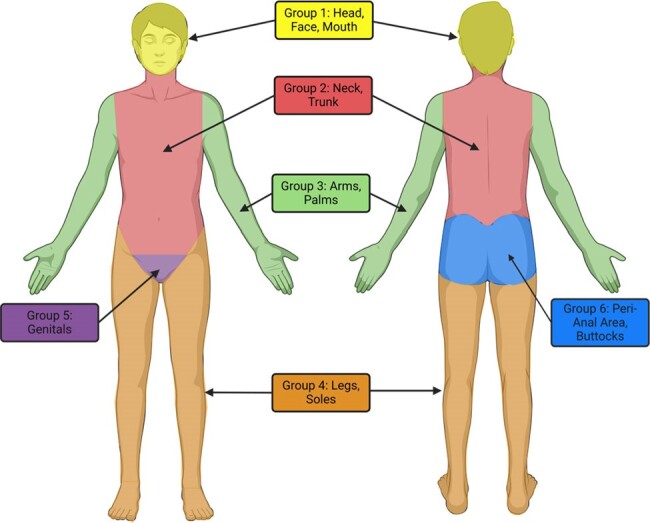

Anatomical regions of the body used to characterize lesion spread. Spread was defined>1 groups with lesions.

**Results:**

Analyses included 4,613 mpox cases; 3,045 (66.0%) reported lesions disseminated across multiple anatomical regions and 1,956 (42.4%) were living with HIV infection. Among cases with disseminated lesions, 114 (3.7%) had received any pre-exposure vaccination and 43 (1.4%) received post-exposure vaccination, compared with 286 (18.2%) and 146 (9.3%), respectively, of 1,568 cases with lesions contained to a single region. VE against progression to disseminated disease was 58.9% (95% confidence interval: 50.4-65.9%) for any pre-exposure immunization with JYNNEOS, and 57.6% (46.3-66.5%) and 61.0% (47.3-71.1%), respectively, for 1 and 2 dose JYNNEOS series. VE against progression to disseminated disease was 15.9% (3.3-26.8%) for cases who received JYNNEOS only as post-exposure prophylaxis. Pre-exposure VE against progression to disseminated disease was 66.4% (56.6-74.0%) for HIV-negative cases and 45.3% (28.0-58.5%) for cases living with HIV.

Proportion of Individuals with Regions Affected by Lesions

**Figure:**
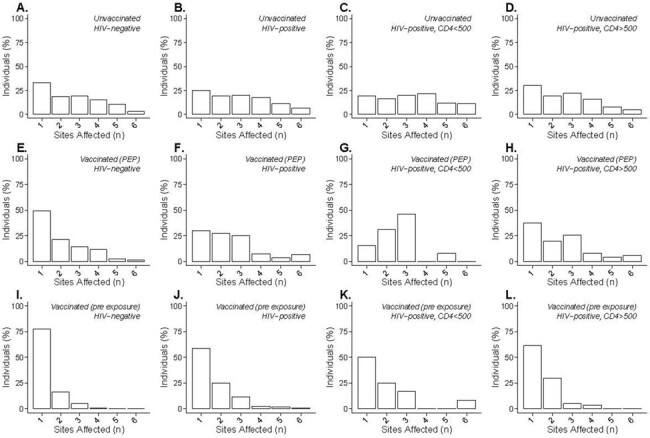

The proportion of mpox cases in California in each subpopulation who reported anywhere from 1-6 body site groups, as defined in figure 2, affected with lesions. A-D represent the unvaccinated population of mpox cases, E-H is the population given PEP, and I-L the vaccinated population (either single or double vaccinated). The first column A, E and I show the HIV- negative population, B, F, and J are persons with HIV (PWH), C, G, and K are PWH with CD4 <500, and D, H, and L are PWH with CD4 ≥ 500.

**Conclusion:**

All persons at risk for mpox should receive JYNNEOS vaccine series to reduce the odds of disseminated disease. Among persons infected with mpox, including those living with HIV, vaccination with JYNNEOS is associated with less severe clinical presentation. Protection was greatest when the vaccine was administered prior to exposure. Strategies are needed to ensure continuous access to JYNNEOS vaccination among all at-risk persons.

VE of JYNNEOS in Preventing Lesion Spread

**Figure:**
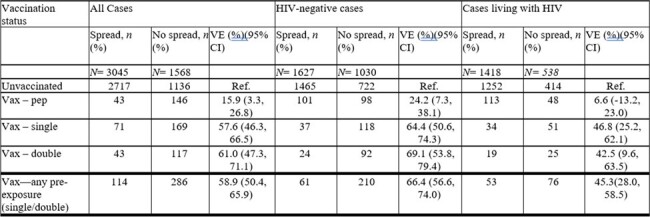

VE of JYNNEOS for PEP, single, double, and any pre-exposure vaccination in reference to a nonvaccinated counterpart. Conditional logistic regression models matching individuals in the “all cases” category on HIV status to estimate VE given number and timing of doses received. Additionally, individuals were stratified by HIV status. All vaccinated groups were calculated in reference to an unvaccinated population.

**Disclosures:**

**All Authors**: No reported disclosures

